# Potassium chlorido­tris­(hypersil­oxy)aluminate dimer

**DOI:** 10.1107/S2056989019005310

**Published:** 2019-05-03

**Authors:** Andrew P. Purdy, Raymond J. Butcher

**Affiliations:** a Chemistry Division, Code 6100, Naval Research Laboratory, 4555 overlook Av, SW, Washington, DC 20375-5342, USA; b Howard University, Chemistry Department, 525 College St NW, Washington, DC 20059, USA

**Keywords:** crystal structure, siloxide

## Abstract

The tris­(tri­methyl­silylsiloxide) ligand, also known as hypersiloxide, is an extremely bulky group. In an attempt to make the monomeric Al(OSi(SiMe_3_)_3_)_3_, AlCl_3_ was combined with 3 equiv. of potassium hypersiloxide. The crystalline product isolated (40% yield), namely di-μ_3_-chlorido-bis­[μ_2_-tris­(tri­methyl­sil­yl)silanolato]tetra­kis­[tris­(tri­methyl­sil­yl)silanolato]dialuminium­dipotassium, [Al_2_K_2_Cl_2_(C_19_H_27_OSi_4_)_6_], is a KCl adduct of aluminium tris(hypersilyloxide) that is dimerized through a planar K_2_Cl_2_ ring, with K—Cl distances of 3.1131 (8) and 3.319 (3) Å, and ring angles of 77.41 (2) and 102.60 (2)°. This ring is on an inversion center, and there is no supra­molecular coordination.

## Abstract 2

The tris­(tri­methyl­silylsiloxide) ligand, also known as hypersiloxide, is an extremely bulky group. In an attempt to make the monomeric Al(OSi(SiMe_3_)_3_)_3_, AlCl_3_ was combined with 3 equiv. of potassium hypersiloxide. The title compound, a KCl adduct of aluminium tris­(hypersilyloxide) that is dimerized through a planar K_2_Cl_2_ ring, was isolated.

## Chemical context   

Alkoxides and siloxides of electropositive metals with empty *p* and *d* orbitals, such as aluminum, tend to be dimers, trimers, or higher oligomers, from coordination between the alk­oxy oxygens and the metal atom on another mol­ecule. One way to prepare monomeric homoleptic compounds of such metals is by using extremely bulky ligands that prevent inter­molecular coordination. One of the bulkiest siloxide ligands known is tris­(tri­methyl­sil­yl)silyl, also known as hypersilyl (Niemeyer, 2006 ▸). While the hypersiloxide (Boyle *et al.*, 2018 ▸) could potentially have enough steric bulk to enable a homoleptic monomeric aluminum alkoxide to be prepared, we isolated a dimer of a KCl addition compound, dimerized through potassium and chlorine. The title compound was the only product that crystallized, but there were other products present, and these products decomposed during an attempt at sublimation. If less bulky, and thus more volatile, aluminum siloxides or alkoxides can form a stable soluble KCl adducts, which could lead to a means of solubilizing alkali metal halides from organometallic reactions without using protic solvents.

## Structural commentary   

**Figure 1:**
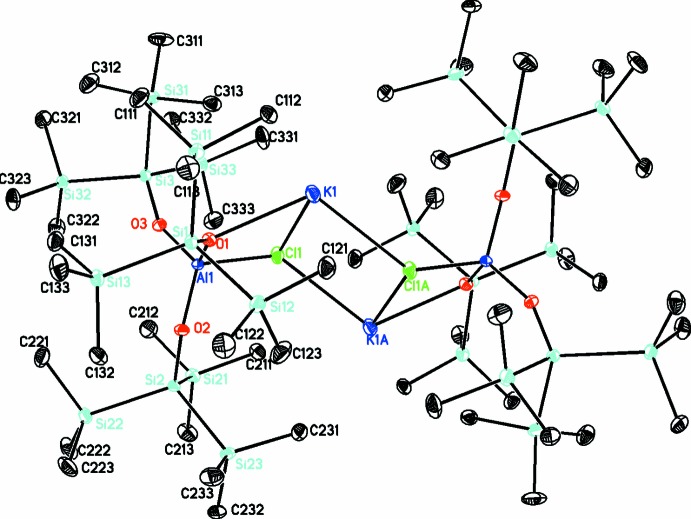
Diagram of the title compound, with displacement ellipsoids drawn at the 30% probability level. H atoms omitted for clarity. Symmetry code A: 1 − *x*, 1 − *y*, 1 − *z*.

Each tris­(hypersil­oxy)chloro­aluminate ion is joined into the dimeric structure by a K^+^ ion coordinated to the chlorine atoms and one of the sil­oxy oxygen atoms, O1 (Fig. 1 ▸). The K_2_Cl_2_ ring is constrained by symmetry to be planar as it is on an inversion center, but the adjoined four-membered K1–Cl1–O1–Al1 rings deviate slightly from planarity, with the angle between the K_2_Cl_2_ plane and mean K1–Cl1–Al1–O1 planes being 47.8 (1)°. The coordination around the aluminum atom is approximately tetra­hedral with angles ranging from 100.32 (6) to 114.63 (8)°. Both Al—O bonds to the terminal sil­oxy ligands are 1.711 (1) Å, and the Al1—O1 bond is slightly longer at 1.746 (1) Å, within the normal range for aluminum siloxides. In a series of aluminum complexes of silanediols, terminal Al—O bonds ranged from 1.709 (2) to 1.781 (4) Å, and all Al—O bonds to siloxide oxygen atoms bridging between aluminum atoms were longer than 1.8 Å (Krempner *et al.*, 2007 ▸). Likewise, the aluminum phenyl­siloxide Al(OSiPh_3_)_3_(THF) with all terminal siloxides has Al—O bond lengths ranging from 1.696 (5) to 1.709 (5) Å (Apblett *et al.* 1992 ▸).

K_2_Cl_2_ rings in organometallic complexes can be isolated or part of larger K—Cl assemblages. For isolated K_2_Cl_2_ rings, both planar and puckered rings are known, with planar rings lying on an inversion center the most common. The K—Cl distances in the title compound at 3.1131 (8) and 3.319 (3) Å are normal for this kind of feature, and the ring angles of 77.41 (2) and 102.60 (2)° around K and Cl, respectively, are typical for this kind of ring. Reported examples of similar features are in K[GaCl]{Co_2_(CO)_6_(μ-CO)}{Co(CO)_4_}, which has K—Cl distances of 3.129 (1) and 3.197 (1) Å and angles of 73.82 (3) and 106.18 (3)° (Leiner *et al.*, 2002 ▸), and in a chloro­aluminate complex [K—Cl distances of 3.160 (2) and 3.192 (1) Å and angles of 75.66 (3) and 104.24 (4)°; Abdalla, *et al.*, 2015 ▸].

## Supra­molecular features   

The mol­ecule is completely surrounded by ligands and thus there are no supra­molecular features.

## Database survey   

A search of the Cambridge Structural Database (V 5.38, update May 2017; Groom *et al.*, 2016 ▸) for Al siloxides produced 255 hits, and the most common moieties have either phenyl or methyl attached to the silicon. However, no aluminum complexes of the tris­(tri­methyl­sil­yl)sil­oxy ligand were found. The closest analogs are some complexes of chelating silanediols HO(Me_3_Si)_2_SiSi(SiMe_3_)_2_OH and HO(Me)[(Me_3_Si)_3_Si]SiSi[Si(SiMe_3_)_3_](Me)OH (Krempner *et al.*, 2007 ▸). A search for a tri(organosil­yl)sil­oxy ligand attached to a metal produced six unique hits. These include Fe^II^ and Co^II^ complexes (Kornev *et al.*, 1997 ▸ and Chesnokova *et al.*, 2002 ▸), lanthanide(III) complexes (Kornev *et al.*, 1999 ▸), and Ta^V^ complexes (Wu *et al.*, 2002 ▸).

## Synthesis and crystallization   

Hypersilanol was prepared by literature methods (Gilman & Harrell, 1966 ▸). The silanol HOSi(SiMe_3_)_3_ (3.00 g, 11.3 mmol) was mixed with KH (0.45 g, 11.2 mmol) and dry heptane (15 mL) in a reaction bulb equipped with a Kontes valve in an argon-filled drybox. After 1 day, sublimed AlCl_3_ (0.50 g, 3.75 mmol) was added to the bulb with another 5 mL of heptane, and the bulb was sonicated for 1 day in a bath sonicator. The reaction was returned to the drybox and filtration was attempted through a fine frit. The frit clogged after a small amount of filtrate went through. Filtration was resumed through 1 cm diameter PTFE membranes with a nominal 0.22 µm size. The membrane had to be changed twice, but filtration was finally completed after two weeks. Crystals of the title compound grew in the filtrate (64 mg isolated). The liquid was deca­nted from the crystals, the solvent removed, leaving a semi-solid mixture. An attempt at sublimation resulted in decomposition.

A repeat preparation using the same qu­anti­ties of reactants in 40 mL heptane was sonicated for 3 h and then stirred at 338 K overnight. The mixture was filtered through a 47 mm diameter 0.22 µm PVDF filter membrane, and the filtrate pumped to a white solid, whose NMR showed multiple products. This white solid was recrystallized from a minimum amount of hot heptane under argon, affording 1.335 g (40%) of colorless crystals of the title compound. NMR (C_6_D_6_): ^1^H δ0.36; ^13^C 3.36; ^29^Si −22.39 (Si), −18.36 (SiMe_3_).

## Refinement   

Crystal data, data collection and structure refinement details are summarized in Table 1 ▸.

## Supplementary Material

Crystal structure: contains datablock(s) I. DOI: 10.1107/S2056989019005310/nk2251sup1.cif

Structure factors: contains datablock(s) I. DOI: 10.1107/S2056989019005310/nk2251Isup2.hkl

CCDC reference: 1910627

Additional supporting information:  crystallographic information; 3D view; checkCIF report

## Figures and Tables

**Table 1:** Experimental details

Crystal data	
Chemical formula	[Al_2_K_2_Cl_2_(C_19_H_27_OSi_4_)_6_]
*M*_r_	1785.05
Crystal system, space group	Monoclinic, *P*2_1_/*n*
Temperature (K)	173
*a*, *b*, *c* (Å)	18.8247 (3), 13.8850 (2), 22.2056 (4)
β (°)	109.413 (2)
*V* (Å^3^)	5474.14 (17)
*Z*	2
Radiation type	Cu *K*α
μ (mm^−1^)	4.16
Crystal size (mm)	0.37 × 0.31 × 0.24

Data collection	
Diffractometer	Agilent Xcalibur Eos Gemini
Absorption correction	Multi-scan (*CrysAlis PRO*; Agilent, 2014 ▸)
*T*_min_, *T*_max_	0.441, 1.000
No. of measured, independent and observed [*I* > 2σ(*I*)] reflections	25914, 10450, 8699
*R*_int_	0.035
(sin θ/λ)_max_ (Å^−1^)	0.615

Refinement	
*R*[*F* ^2^ > 2σ(*F* ^2^)], *wR*(*F* ^2^), *S*	0.042, 0.117, 1.04
No. of reflections	10450
No. of parameters	433
H-atom treatment	H-atom parameters constrained
Δρ_max_, Δρ_min_ (e Å^−3^)	0.76, −0.28

Computer programs: *CrysAlis PRO* (Agilent, 2014 ▸), *SHELXT* (Sheldrick, 2015*a* ▸), *SHELXL2016* (Sheldrick, 2015*b* ▸) and *SHELXTL* (Sheldrick, 2008 ▸).

## supplementary crystallographic information

### Crystal data

**Table:**

[Al_2_K_2_Cl_2_(C_19_H_27_OSi_4_)_6_]	*F*(000) = 1936
*M**_r_* = 1785.05	*D*_x_ = 1.083 Mg m^−^^3^
Monoclinic, *P*2_1_/*n*	Cu *K*α radiation, λ = 1.54184 Å
*a* = 18.8247 (3) Å	Cell parameters from 9927 reflections
*b* = 13.8850 (2) Å	θ = 4.0–71.3°
*c* = 22.2056 (4) Å	µ = 4.16 mm^−^^1^
β = 109.413 (2)°	*T* = 173 K
*V* = 5474.14 (17) Å^3^	Block, colorless
*Z* = 2	0.37 × 0.31 × 0.24 mm

### Data collection

**Table:**

Agilent Xcalibur Eos Gemini diffractometer	10450 independent reflections
Radiation source: Enhance (Cu) X-ray Source	8699 reflections with *I* > 2σ(*I*)
Detector resolution: 16.0416 pixels mm^-1^	*R*_int_ = 0.035
ω scans	θ_max_ = 71.4°, θ_min_ = 3.8°
Absorption correction: multi-scan (CrysAlis PRO; Agilent, 2014)	*h* = −23→19
*T*_min_ = 0.441, *T*_max_ = 1.000	*k* = −16→16
25914 measured reflections	*l* = −27→25

### Refinement

**Table:**

Refinement on *F*^2^	0 restraints
Least-squares matrix: full	Hydrogen site location: inferred from neighbouring sites
*R*[*F*^2^ > 2σ(*F*^2^)] = 0.042	H-atom parameters constrained
*wR*(*F*^2^) = 0.117	*w* = 1/[σ^2^(*F*_o_^2^) + (0.0711*P*)^2^ + 0.624*P*] where *P* = (*F*_o_^2^ + 2*F*_c_^2^)/3
*S* = 1.04	(Δ/σ)_max_ = 0.001
10450 reflections	Δρ_max_ = 0.76 e Å^−^^3^
433 parameters	Δρ_min_ = −0.28 e Å^−^^3^

### Special details

**Table:**

Geometry. All esds (except the esd in the dihedral angle between two l.s. planes) are estimated using the full covariance matrix. The cell esds are taken into account individually in the estimation of esds in distances, angles and torsion angles; correlations between esds in cell parameters are only used when they are defined by crystal symmetry. An approximate (isotropic) treatment of cell esds is used for estimating esds involving l.s. planes.

### Fractional atomic coordinates and isotropic or equivalent isotropic displacement parameters (Å^2^)

**Table:**

	*x*	*y*	*z*	*U*_iso_*/*U*_eq_	
K1	0.57420 (3)	0.37104 (4)	0.48201 (3)	0.04295 (14)	
Cl1	0.47952 (3)	0.53645 (4)	0.41286 (3)	0.03676 (13)	
Si1	0.73143 (3)	0.46759 (4)	0.44589 (3)	0.02465 (12)	
Si2	0.57625 (3)	0.80409 (4)	0.38317 (3)	0.02508 (13)	
Si3	0.47806 (3)	0.47751 (4)	0.23042 (3)	0.02570 (13)	
Si11	0.76601 (4)	0.30398 (5)	0.44632 (3)	0.03327 (14)	
Si12	0.77130 (4)	0.51357 (5)	0.55459 (3)	0.03630 (15)	
Si13	0.79768 (3)	0.55732 (5)	0.39166 (3)	0.03275 (14)	
Si21	0.44683 (3)	0.83662 (5)	0.33310 (3)	0.03364 (14)	
Si22	0.63846 (4)	0.88865 (5)	0.32264 (4)	0.04016 (16)	
Si23	0.61445 (4)	0.86915 (5)	0.48644 (3)	0.03486 (15)	
Si31	0.52190 (4)	0.31699 (5)	0.23980 (3)	0.03842 (16)	
Si32	0.48618 (3)	0.54112 (5)	0.13426 (3)	0.03018 (14)	
Si33	0.34999 (4)	0.46420 (5)	0.22239 (3)	0.03741 (16)	
Al1	0.56972 (3)	0.56860 (4)	0.37091 (3)	0.02094 (13)	
O1	0.63984 (8)	0.48937 (10)	0.41485 (7)	0.0261 (3)	
O2	0.59216 (8)	0.68797 (10)	0.38410 (7)	0.0278 (3)	
O3	0.53047 (8)	0.53867 (11)	0.29203 (7)	0.0288 (3)	
C111	0.75888 (18)	0.2512 (2)	0.36675 (14)	0.0534 (7)	
H11A	0.780011	0.186011	0.372780	0.080*	
H11B	0.787022	0.291510	0.346281	0.080*	
H11C	0.705870	0.248551	0.339614	0.080*	
C112	0.71162 (17)	0.22111 (19)	0.48223 (13)	0.0451 (6)	
H11D	0.735733	0.157629	0.489608	0.068*	
H11E	0.659848	0.214767	0.452859	0.068*	
H11F	0.710990	0.247837	0.522896	0.068*	
C113	0.86772 (16)	0.2993 (2)	0.49849 (15)	0.0540 (7)	
H11G	0.884887	0.232238	0.503648	0.081*	
H11H	0.873035	0.326784	0.540422	0.081*	
H11I	0.898232	0.336493	0.478592	0.081*	
C121	0.76369 (18)	0.4170 (2)	0.61159 (12)	0.0530 (7)	
H12A	0.779474	0.443114	0.655056	0.080*	
H12B	0.796250	0.362763	0.609887	0.080*	
H12C	0.711370	0.394934	0.599518	0.080*	
C122	0.8713 (2)	0.5559 (3)	0.58315 (15)	0.0752 (12)	
H12D	0.884643	0.578207	0.627406	0.113*	
H12E	0.877071	0.609061	0.556098	0.113*	
H12F	0.904521	0.502731	0.580911	0.113*	
C123	0.7030 (3)	0.6105 (3)	0.55869 (17)	0.0743 (11)	
H12G	0.724758	0.648099	0.597890	0.111*	
H12H	0.655816	0.580877	0.558750	0.111*	
H12I	0.693022	0.653051	0.521565	0.111*	
C131	0.74560 (17)	0.5443 (2)	0.30394 (12)	0.0478 (6)	
H13A	0.767512	0.587768	0.280079	0.072*	
H13B	0.692443	0.560660	0.295038	0.072*	
H13C	0.749622	0.477677	0.290865	0.072*	
C132	0.79786 (18)	0.6873 (2)	0.41371 (15)	0.0513 (7)	
H13D	0.818651	0.725943	0.386573	0.077*	
H13E	0.828843	0.696002	0.458561	0.077*	
H13F	0.746187	0.708185	0.407553	0.077*	
C133	0.89776 (16)	0.5175 (3)	0.40851 (17)	0.0598 (8)	
H13G	0.922440	0.560244	0.386404	0.090*	
H13H	0.898381	0.451353	0.393329	0.090*	
H13I	0.924675	0.520185	0.454585	0.090*	
C211	0.38795 (14)	0.77173 (19)	0.37411 (14)	0.0448 (6)	
H21A	0.334764	0.775116	0.347502	0.067*	
H21B	0.394737	0.802112	0.415485	0.067*	
H21C	0.403673	0.704162	0.380699	0.067*	
C212	0.42149 (18)	0.7911 (2)	0.24922 (13)	0.0543 (7)	
H21D	0.367071	0.797614	0.227717	0.082*	
H21E	0.435827	0.723211	0.249816	0.082*	
H21F	0.448313	0.828807	0.226233	0.082*	
C213	0.41897 (17)	0.9676 (2)	0.33006 (18)	0.0574 (8)	
H21G	0.367020	0.975242	0.301292	0.086*	
H21H	0.452743	1.006107	0.314321	0.086*	
H21I	0.422672	0.989346	0.372975	0.086*	
C221	0.6506 (2)	0.8050 (2)	0.26151 (17)	0.0658 (9)	
H22A	0.673766	0.839512	0.234291	0.099*	
H22B	0.601304	0.780109	0.235263	0.099*	
H22C	0.683072	0.751315	0.282708	0.099*	
C222	0.5826 (2)	0.9958 (2)	0.2800 (2)	0.0682 (10)	
H22D	0.610576	1.028923	0.255949	0.102*	
H22E	0.574274	1.040142	0.311312	0.102*	
H22F	0.533950	0.974001	0.250513	0.102*	
C223	0.73237 (18)	0.9395 (2)	0.37176 (19)	0.0645 (9)	
H22G	0.757250	0.967287	0.343418	0.097*	
H22H	0.763844	0.888025	0.397332	0.097*	
H22I	0.724997	0.989669	0.400156	0.097*	
C231	0.56436 (17)	0.8067 (2)	0.53611 (13)	0.0477 (6)	
H23A	0.584952	0.829141	0.580392	0.072*	
H23B	0.571624	0.736987	0.534685	0.072*	
H23C	0.510450	0.821594	0.519109	0.072*	
C232	0.5892 (2)	1.00077 (19)	0.47939 (16)	0.0545 (7)	
H23D	0.606557	1.030377	0.521865	0.082*	
H23E	0.534465	1.007830	0.460654	0.082*	
H23F	0.613525	1.032639	0.451989	0.082*	
C233	0.71955 (18)	0.8631 (3)	0.52913 (16)	0.0627 (8)	
H23G	0.730299	0.874770	0.574827	0.094*	
H23H	0.744673	0.912297	0.511712	0.094*	
H23I	0.738178	0.799260	0.522965	0.094*	
C311	0.4504 (2)	0.2270 (2)	0.19217 (15)	0.0671 (10)	
H31A	0.474201	0.163627	0.194905	0.101*	
H31B	0.430846	0.247478	0.147429	0.101*	
H31C	0.408789	0.222938	0.209386	0.101*	
C312	0.6072 (2)	0.3029 (2)	0.21447 (15)	0.0596 (8)	
H31D	0.628708	0.238580	0.226263	0.089*	
H31E	0.644708	0.351717	0.235836	0.089*	
H31F	0.592593	0.311132	0.168107	0.089*	
C313	0.54652 (17)	0.28382 (19)	0.32684 (12)	0.0446 (6)	
H31G	0.564946	0.217323	0.333273	0.067*	
H31H	0.501596	0.289644	0.339574	0.067*	
H31I	0.585781	0.327295	0.352851	0.067*	
C321	0.4486 (2)	0.4501 (2)	0.06848 (13)	0.0594 (8)	
H32A	0.455440	0.474287	0.029288	0.089*	
H32B	0.394937	0.439418	0.061008	0.089*	
H32C	0.476025	0.389309	0.080909	0.089*	
C322	0.43252 (19)	0.6563 (2)	0.10763 (14)	0.0564 (7)	
H32D	0.436173	0.675891	0.066364	0.085*	
H32E	0.453887	0.706765	0.139331	0.085*	
H32F	0.379520	0.646261	0.103305	0.085*	
C323	0.58614 (15)	0.5661 (3)	0.14213 (14)	0.0519 (7)	
H32G	0.588707	0.589139	0.101178	0.078*	
H32H	0.615790	0.506953	0.154461	0.078*	
H32I	0.606491	0.615507	0.174890	0.078*	
C331	0.33467 (19)	0.3778 (3)	0.28202 (14)	0.0657 (10)	
H33A	0.280583	0.371597	0.274680	0.099*	
H33B	0.360012	0.402047	0.325314	0.099*	
H33C	0.355427	0.314680	0.277160	0.099*	
C332	0.29642 (15)	0.4164 (2)	0.14070 (12)	0.0471 (6)	
H33D	0.242748	0.412544	0.135569	0.071*	
H33E	0.315193	0.352144	0.135671	0.071*	
H33F	0.303400	0.459714	0.108249	0.071*	
C333	0.30521 (16)	0.5810 (3)	0.23174 (17)	0.0604 (8)	
H33G	0.252048	0.570193	0.226489	0.091*	
H33H	0.309020	0.626685	0.199285	0.091*	
H33I	0.331125	0.607448	0.274338	0.091*	

### Atomic displacement parameters (Å^2^)

**Table:**

	*U*^11^	*U*^22^	*U*^33^	*U*^12^	*U*^13^	*U*^23^
K1	0.0524 (3)	0.0352 (3)	0.0494 (3)	0.0059 (2)	0.0278 (3)	0.0117 (2)
Cl1	0.0353 (3)	0.0404 (3)	0.0420 (3)	0.0060 (2)	0.0228 (2)	0.0082 (2)
Si1	0.0240 (3)	0.0245 (3)	0.0269 (3)	0.0041 (2)	0.0104 (2)	0.0041 (2)
Si2	0.0270 (3)	0.0184 (3)	0.0336 (3)	−0.0010 (2)	0.0153 (2)	−0.0031 (2)
Si3	0.0269 (3)	0.0277 (3)	0.0226 (3)	−0.0050 (2)	0.0083 (2)	−0.0025 (2)
Si11	0.0381 (3)	0.0288 (3)	0.0358 (3)	0.0127 (2)	0.0162 (3)	0.0085 (2)
Si12	0.0408 (3)	0.0391 (4)	0.0291 (3)	0.0003 (3)	0.0117 (3)	−0.0002 (3)
Si13	0.0298 (3)	0.0355 (3)	0.0379 (3)	0.0023 (2)	0.0178 (2)	0.0055 (3)
Si21	0.0303 (3)	0.0282 (3)	0.0436 (3)	0.0052 (2)	0.0138 (3)	0.0051 (3)
Si22	0.0499 (4)	0.0252 (3)	0.0597 (4)	0.0017 (3)	0.0375 (3)	0.0051 (3)
Si23	0.0404 (3)	0.0271 (3)	0.0401 (3)	−0.0048 (2)	0.0175 (3)	−0.0115 (3)
Si31	0.0528 (4)	0.0270 (3)	0.0310 (3)	−0.0010 (3)	0.0079 (3)	−0.0054 (2)
Si32	0.0312 (3)	0.0366 (3)	0.0236 (3)	−0.0053 (2)	0.0103 (2)	−0.0019 (2)
Si33	0.0304 (3)	0.0495 (4)	0.0332 (3)	−0.0151 (3)	0.0118 (2)	−0.0052 (3)
Al1	0.0223 (3)	0.0180 (3)	0.0226 (3)	0.0010 (2)	0.0075 (2)	−0.0008 (2)
O1	0.0240 (7)	0.0234 (7)	0.0308 (7)	0.0039 (5)	0.0088 (6)	0.0026 (6)
O2	0.0302 (7)	0.0190 (7)	0.0343 (7)	−0.0003 (6)	0.0108 (6)	−0.0037 (6)
O3	0.0320 (7)	0.0294 (8)	0.0238 (7)	−0.0048 (6)	0.0075 (6)	−0.0048 (6)
C111	0.0675 (18)	0.0499 (17)	0.0515 (15)	0.0184 (14)	0.0315 (14)	−0.0021 (13)
C112	0.0609 (16)	0.0307 (13)	0.0491 (14)	0.0054 (11)	0.0257 (12)	0.0119 (11)
C113	0.0431 (14)	0.0533 (17)	0.0627 (17)	0.0198 (13)	0.0135 (13)	0.0171 (14)
C121	0.0633 (17)	0.0625 (19)	0.0324 (12)	0.0003 (15)	0.0146 (12)	0.0096 (13)
C122	0.065 (2)	0.107 (3)	0.0441 (16)	−0.042 (2)	0.0053 (15)	−0.0160 (18)
C123	0.118 (3)	0.058 (2)	0.0582 (19)	0.032 (2)	0.045 (2)	0.0017 (16)
C131	0.0582 (16)	0.0527 (16)	0.0373 (13)	0.0033 (13)	0.0224 (12)	0.0050 (12)
C132	0.0608 (17)	0.0366 (14)	0.0649 (17)	−0.0077 (12)	0.0322 (14)	0.0028 (13)
C133	0.0366 (14)	0.076 (2)	0.075 (2)	0.0113 (14)	0.0303 (14)	0.0167 (17)
C211	0.0378 (12)	0.0379 (14)	0.0663 (17)	0.0050 (10)	0.0275 (12)	0.0052 (12)
C212	0.0565 (16)	0.0589 (18)	0.0407 (14)	−0.0038 (14)	0.0069 (12)	0.0012 (13)
C213	0.0492 (16)	0.0353 (15)	0.090 (2)	0.0140 (12)	0.0258 (16)	0.0115 (14)
C221	0.099 (3)	0.0534 (19)	0.070 (2)	0.0097 (17)	0.061 (2)	0.0019 (16)
C222	0.081 (2)	0.0392 (16)	0.105 (3)	0.0161 (15)	0.058 (2)	0.0314 (17)
C223	0.0545 (17)	0.0474 (17)	0.105 (3)	−0.0157 (14)	0.0445 (18)	0.0011 (17)
C231	0.0620 (16)	0.0406 (14)	0.0496 (14)	0.0032 (12)	0.0305 (13)	−0.0003 (12)
C232	0.078 (2)	0.0283 (13)	0.0685 (18)	−0.0056 (13)	0.0391 (16)	−0.0143 (13)
C233	0.0481 (16)	0.066 (2)	0.0626 (19)	−0.0140 (15)	0.0029 (14)	−0.0167 (16)
C311	0.093 (3)	0.0316 (14)	0.0540 (17)	−0.0107 (15)	−0.0057 (16)	−0.0135 (13)
C312	0.077 (2)	0.0561 (19)	0.0505 (16)	0.0246 (16)	0.0274 (15)	0.0020 (14)
C313	0.0589 (16)	0.0329 (13)	0.0356 (12)	−0.0020 (11)	0.0071 (11)	0.0028 (10)
C321	0.080 (2)	0.066 (2)	0.0346 (13)	−0.0285 (17)	0.0223 (14)	−0.0183 (13)
C322	0.0623 (18)	0.0596 (19)	0.0481 (15)	0.0176 (15)	0.0195 (14)	0.0193 (14)
C323	0.0360 (13)	0.076 (2)	0.0490 (15)	−0.0073 (13)	0.0212 (11)	0.0049 (14)
C331	0.0564 (17)	0.097 (3)	0.0460 (15)	−0.0347 (18)	0.0200 (13)	0.0073 (16)
C332	0.0406 (13)	0.0558 (17)	0.0375 (12)	−0.0154 (12)	0.0033 (10)	−0.0077 (12)
C333	0.0378 (14)	0.071 (2)	0.077 (2)	−0.0106 (14)	0.0254 (14)	−0.0253 (17)

### Geometric parameters (Å, º)

**Table:**

K1—O1	2.7703 (16)	C123—H12G	0.9800
K1—Cl1	2.9984 (8)	C123—H12H	0.9800
K1—Cl1^i^	3.1131 (8)	C123—H12I	0.9800
K1—C112	3.319 (3)	C131—H13A	0.9800
K1—C231^i^	3.516 (3)	C131—H13B	0.9800
K1—C313	3.524 (3)	C131—H13C	0.9800
K1—Si1	3.5747 (8)	C132—H13D	0.9800
K1—Al1	3.6714 (8)	C132—H13E	0.9800
K1—K1^i^	4.7699 (11)	C132—H13F	0.9800
Cl1—Al1	2.2366 (7)	C133—H13G	0.9800
Si1—O1	1.6581 (14)	C133—H13H	0.9800
Si1—Si13	2.3573 (8)	C133—H13I	0.9800
Si1—Si11	2.3623 (8)	C211—H21A	0.9800
Si1—Si12	2.3648 (8)	C211—H21B	0.9800
Si2—O2	1.6387 (15)	C211—H21C	0.9800
Si2—Si23	2.3443 (8)	C212—H21D	0.9800
Si2—Si21	2.3612 (8)	C212—H21E	0.9800
Si2—Si22	2.3670 (8)	C212—H21F	0.9800
Si3—O3	1.6337 (15)	C213—H21G	0.9800
Si3—Si31	2.3617 (9)	C213—H21H	0.9800
Si3—Si32	2.3623 (8)	C213—H21I	0.9800
Si3—Si33	2.3648 (8)	C221—H22A	0.9800
Si11—C111	1.876 (3)	C221—H22B	0.9800
Si11—C113	1.881 (3)	C221—H22C	0.9800
Si11—C112	1.884 (3)	C222—H22D	0.9800
Si12—C122	1.870 (3)	C222—H22E	0.9800
Si12—C121	1.882 (3)	C222—H22F	0.9800
Si12—C123	1.884 (3)	C223—H22G	0.9800
Si13—C132	1.870 (3)	C223—H22H	0.9800
Si13—C131	1.876 (3)	C223—H22I	0.9800
Si13—C133	1.878 (3)	C231—H23A	0.9800
Si21—C212	1.872 (3)	C231—H23B	0.9800
Si21—C211	1.882 (3)	C231—H23C	0.9800
Si21—C213	1.887 (3)	C232—H23D	0.9800
Si22—C221	1.857 (3)	C232—H23E	0.9800
Si22—C223	1.879 (3)	C232—H23F	0.9800
Si22—C222	1.885 (3)	C233—H23G	0.9800
Si23—C232	1.882 (3)	C233—H23H	0.9800
Si23—C231	1.884 (3)	C233—H23I	0.9800
Si23—C233	1.890 (3)	C311—H31A	0.9800
Si31—C312	1.879 (3)	C311—H31B	0.9800
Si31—C311	1.884 (3)	C311—H31C	0.9800
Si31—C313	1.889 (3)	C312—H31D	0.9800
Si32—C323	1.864 (3)	C312—H31E	0.9800
Si32—C322	1.879 (3)	C312—H31F	0.9800
Si32—C321	1.882 (3)	C313—H31G	0.9800
Si33—C333	1.871 (3)	C313—H31H	0.9800
Si33—C331	1.879 (3)	C313—H31I	0.9800
Si33—C332	1.879 (2)	C321—H32A	0.9800
Al1—O3	1.7107 (14)	C321—H32B	0.9800
Al1—O2	1.7115 (15)	C321—H32C	0.9800
Al1—O1	1.7459 (15)	C322—H32D	0.9800
C111—H11A	0.9800	C322—H32E	0.9800
C111—H11B	0.9800	C322—H32F	0.9800
C111—H11C	0.9800	C323—H32G	0.9800
C112—H11D	0.9800	C323—H32H	0.9800
C112—H11E	0.9800	C323—H32I	0.9800
C112—H11F	0.9800	C331—H33A	0.9800
C113—H11G	0.9800	C331—H33B	0.9800
C113—H11H	0.9800	C331—H33C	0.9800
C113—H11I	0.9800	C332—H33D	0.9800
C121—H12A	0.9800	C332—H33E	0.9800
C121—H12B	0.9800	C332—H33F	0.9800
C121—H12C	0.9800	C333—H33G	0.9800
C122—H12D	0.9800	C333—H33H	0.9800
C122—H12E	0.9800	C333—H33I	0.9800
C122—H12F	0.9800		
			
O1—K1—Cl1	64.26 (3)	Si12—C121—H12A	109.5
O1—K1—Cl1^i^	118.23 (4)	Si12—C121—H12B	109.5
Cl1—K1—Cl1^i^	77.41 (2)	H12A—C121—H12B	109.5
O1—K1—C112	83.45 (5)	Si12—C121—H12C	109.5
Cl1—K1—C112	144.25 (5)	H12A—C121—H12C	109.5
Cl1^i^—K1—C112	134.82 (5)	H12B—C121—H12C	109.5
O1—K1—C231^i^	143.27 (6)	Si12—C122—H12D	109.5
Cl1—K1—C231^i^	100.78 (5)	Si12—C122—H12E	109.5
Cl1^i^—K1—C231^i^	87.82 (5)	H12D—C122—H12E	109.5
C112—K1—C231^i^	96.20 (7)	Si12—C122—H12F	109.5
O1—K1—C313	67.97 (6)	H12D—C122—H12F	109.5
Cl1—K1—C313	83.86 (5)	H12E—C122—H12F	109.5
Cl1^i^—K1—C313	153.60 (5)	Si12—C123—H12G	109.5
C112—K1—C313	69.38 (7)	Si12—C123—H12H	109.5
C231^i^—K1—C313	77.51 (7)	H12G—C123—H12H	109.5
O1—K1—Si1	26.64 (3)	Si12—C123—H12I	109.5
Cl1—K1—Si1	89.626 (18)	H12G—C123—H12I	109.5
Cl1^i^—K1—Si1	120.87 (2)	H12H—C123—H12I	109.5
C112—K1—Si1	62.13 (5)	Si13—C131—H13A	109.5
C231^i^—K1—Si1	151.13 (5)	Si13—C131—H13B	109.5
C313—K1—Si1	76.93 (5)	H13A—C131—H13B	109.5
O1—K1—Al1	27.12 (3)	Si13—C131—H13C	109.5
Cl1—K1—Al1	37.498 (14)	H13A—C131—H13C	109.5
Cl1^i^—K1—Al1	104.77 (2)	H13B—C131—H13C	109.5
C112—K1—Al1	108.27 (5)	Si13—C132—H13D	109.5
C231^i^—K1—Al1	126.67 (5)	Si13—C132—H13E	109.5
C313—K1—Al1	68.71 (4)	H13D—C132—H13E	109.5
Si1—K1—Al1	53.478 (14)	Si13—C132—H13F	109.5
O1—K1—K1^i^	92.05 (3)	H13D—C132—H13F	109.5
Cl1—K1—K1^i^	39.564 (13)	H13E—C132—H13F	109.5
Cl1^i^—K1—K1^i^	37.842 (13)	Si13—C133—H13G	109.5
C112—K1—K1^i^	165.98 (6)	Si13—C133—H13H	109.5
C231^i^—K1—K1^i^	95.32 (5)	H13G—C133—H13H	109.5
C313—K1—K1^i^	121.15 (5)	Si13—C133—H13I	109.5
Si1—K1—K1^i^	109.31 (2)	H13G—C133—H13I	109.5
Al1—K1—K1^i^	70.590 (15)	H13H—C133—H13I	109.5
Al1—Cl1—K1	87.81 (2)	Si21—C211—H21A	109.5
Al1—Cl1—K1^i^	135.30 (3)	Si21—C211—H21B	109.5
K1—Cl1—K1^i^	102.60 (2)	H21A—C211—H21B	109.5
O1—Si1—Si13	110.18 (6)	Si21—C211—H21C	109.5
O1—Si1—Si11	115.10 (6)	H21A—C211—H21C	109.5
Si13—Si1—Si11	108.57 (3)	H21B—C211—H21C	109.5
O1—Si1—Si12	107.96 (6)	Si21—C212—H21D	109.5
Si13—Si1—Si12	109.66 (3)	Si21—C212—H21E	109.5
Si11—Si1—Si12	105.19 (3)	H21D—C212—H21E	109.5
O1—Si1—K1	48.51 (5)	Si21—C212—H21F	109.5
Si13—Si1—K1	158.58 (3)	H21D—C212—H21F	109.5
Si11—Si1—K1	83.46 (2)	H21E—C212—H21F	109.5
Si12—Si1—K1	82.98 (2)	Si21—C213—H21G	109.5
O2—Si2—Si23	111.81 (6)	Si21—C213—H21H	109.5
O2—Si2—Si21	110.47 (6)	H21G—C213—H21H	109.5
Si23—Si2—Si21	108.66 (3)	Si21—C213—H21I	109.5
O2—Si2—Si22	111.86 (6)	H21G—C213—H21I	109.5
Si23—Si2—Si22	108.39 (3)	H21H—C213—H21I	109.5
Si21—Si2—Si22	105.39 (3)	Si22—C221—H22A	109.5
O3—Si3—Si31	108.71 (6)	Si22—C221—H22B	109.5
O3—Si3—Si32	111.10 (6)	H22A—C221—H22B	109.5
Si31—Si3—Si32	107.87 (3)	Si22—C221—H22C	109.5
O3—Si3—Si33	114.87 (6)	H22A—C221—H22C	109.5
Si31—Si3—Si33	104.51 (3)	H22B—C221—H22C	109.5
Si32—Si3—Si33	109.37 (3)	Si22—C222—H22D	109.5
C111—Si11—C113	107.83 (14)	Si22—C222—H22E	109.5
C111—Si11—C112	106.33 (14)	H22D—C222—H22E	109.5
C113—Si11—C112	107.66 (13)	Si22—C222—H22F	109.5
C111—Si11—Si1	116.00 (10)	H22D—C222—H22F	109.5
C113—Si11—Si1	104.97 (10)	H22E—C222—H22F	109.5
C112—Si11—Si1	113.65 (9)	Si22—C223—H22G	109.5
C122—Si12—C121	106.80 (16)	Si22—C223—H22H	109.5
C122—Si12—C123	112.6 (2)	H22G—C223—H22H	109.5
C121—Si12—C123	105.71 (16)	Si22—C223—H22I	109.5
C122—Si12—Si1	111.92 (11)	H22G—C223—H22I	109.5
C121—Si12—Si1	115.15 (10)	H22H—C223—H22I	109.5
C123—Si12—Si1	104.60 (12)	Si23—C231—K1^i^	138.83 (13)
C132—Si13—C131	108.65 (14)	Si23—C231—H23A	109.5
C132—Si13—C133	108.52 (16)	K1^i^—C231—H23A	111.6
C131—Si13—C133	108.79 (14)	Si23—C231—H23B	109.5
C132—Si13—Si1	109.36 (9)	K1^i^—C231—H23B	53.8
C131—Si13—Si1	107.27 (10)	H23A—C231—H23B	109.5
C133—Si13—Si1	114.13 (11)	Si23—C231—H23C	109.5
C212—Si21—C211	108.89 (14)	K1^i^—C231—H23C	58.0
C212—Si21—C213	108.26 (16)	H23A—C231—H23C	109.5
C211—Si21—C213	106.10 (13)	H23B—C231—H23C	109.5
C212—Si21—Si2	106.61 (11)	Si23—C232—H23D	109.5
C211—Si21—Si2	111.23 (9)	Si23—C232—H23E	109.5
C213—Si21—Si2	115.60 (10)	H23D—C232—H23E	109.5
C221—Si22—C223	109.79 (17)	Si23—C232—H23F	109.5
C221—Si22—C222	108.03 (17)	H23D—C232—H23F	109.5
C223—Si22—C222	104.79 (17)	H23E—C232—H23F	109.5
C221—Si22—Si2	108.00 (11)	Si23—C233—H23G	109.5
C223—Si22—Si2	113.84 (12)	Si23—C233—H23H	109.5
C222—Si22—Si2	112.23 (11)	H23G—C233—H23H	109.5
C232—Si23—C231	109.51 (13)	Si23—C233—H23I	109.5
C232—Si23—C233	106.12 (15)	H23G—C233—H23I	109.5
C231—Si23—C233	109.29 (15)	H23H—C233—H23I	109.5
C232—Si23—Si2	107.82 (11)	Si31—C311—H31A	109.5
C231—Si23—Si2	110.18 (9)	Si31—C311—H31B	109.5
C233—Si23—Si2	113.77 (11)	H31A—C311—H31B	109.5
C312—Si31—C311	106.59 (17)	Si31—C311—H31C	109.5
C312—Si31—C313	109.72 (14)	H31A—C311—H31C	109.5
C311—Si31—C313	107.41 (14)	H31B—C311—H31C	109.5
C312—Si31—Si3	112.33 (11)	Si31—C312—H31D	109.5
C311—Si31—Si3	114.40 (11)	Si31—C312—H31E	109.5
C313—Si31—Si3	106.26 (9)	H31D—C312—H31E	109.5
C323—Si32—C322	106.93 (15)	Si31—C312—H31F	109.5
C323—Si32—C321	107.74 (15)	H31D—C312—H31F	109.5
C322—Si32—C321	107.65 (16)	H31E—C312—H31F	109.5
C323—Si32—Si3	110.59 (9)	Si31—C313—K1	145.40 (12)
C322—Si32—Si3	114.41 (10)	Si31—C313—H31G	109.5
C321—Si32—Si3	109.27 (10)	K1—C313—H31G	104.6
C333—Si33—C331	107.17 (17)	Si31—C313—H31H	109.5
C333—Si33—C332	107.22 (14)	K1—C313—H31H	63.3
C331—Si33—C332	107.23 (14)	H31G—C313—H31H	109.5
C333—Si33—Si3	114.17 (10)	Si31—C313—H31I	109.5
C331—Si33—Si3	112.84 (11)	K1—C313—H31I	51.3
C332—Si33—Si3	107.87 (9)	H31G—C313—H31I	109.5
O3—Al1—O2	114.11 (8)	H31H—C313—H31I	109.5
O3—Al1—O1	114.09 (8)	Si32—C321—H32A	109.5
O2—Al1—O1	114.64 (7)	Si32—C321—H32B	109.5
O3—Al1—Cl1	104.39 (6)	H32A—C321—H32B	109.5
O2—Al1—Cl1	107.48 (6)	Si32—C321—H32C	109.5
O1—Al1—Cl1	100.32 (6)	H32A—C321—H32C	109.5
O3—Al1—K1	114.34 (6)	H32B—C321—H32C	109.5
O2—Al1—K1	131.25 (6)	Si32—C322—H32D	109.5
O1—Al1—K1	46.33 (5)	Si32—C322—H32E	109.5
Cl1—Al1—K1	54.69 (2)	H32D—C322—H32E	109.5
Si1—O1—Al1	146.69 (10)	Si32—C322—H32F	109.5
Si1—O1—K1	104.85 (7)	H32D—C322—H32F	109.5
Al1—O1—K1	106.56 (6)	H32E—C322—H32F	109.5
Si2—O2—Al1	156.49 (10)	Si32—C323—H32G	109.5
Si3—O3—Al1	156.75 (10)	Si32—C323—H32H	109.5
Si11—C111—H11A	109.5	H32G—C323—H32H	109.5
Si11—C111—H11B	109.5	Si32—C323—H32I	109.5
H11A—C111—H11B	109.5	H32G—C323—H32I	109.5
Si11—C111—H11C	109.5	H32H—C323—H32I	109.5
H11A—C111—H11C	109.5	Si33—C331—H33A	109.5
H11B—C111—H11C	109.5	Si33—C331—H33B	109.5
Si11—C112—K1	98.58 (10)	H33A—C331—H33B	109.5
Si11—C112—H11D	109.5	Si33—C331—H33C	109.5
K1—C112—H11D	151.9	H33A—C331—H33C	109.5
Si11—C112—H11E	109.5	H33B—C331—H33C	109.5
K1—C112—H11E	56.9	Si33—C332—H33D	109.5
H11D—C112—H11E	109.5	Si33—C332—H33E	109.5
Si11—C112—H11F	109.5	H33D—C332—H33E	109.5
K1—C112—H11F	61.0	Si33—C332—H33F	109.5
H11D—C112—H11F	109.5	H33D—C332—H33F	109.5
H11E—C112—H11F	109.5	H33E—C332—H33F	109.5
Si11—C113—H11G	109.5	Si33—C333—H33G	109.5
Si11—C113—H11H	109.5	Si33—C333—H33H	109.5
H11G—C113—H11H	109.5	H33G—C333—H33H	109.5
Si11—C113—H11I	109.5	Si33—C333—H33I	109.5
H11G—C113—H11I	109.5	H33G—C333—H33I	109.5
H11H—C113—H11I	109.5	H33H—C333—H33I	109.5
			
Si13—Si1—O1—Al1	−22.36 (19)	Cl1—Al1—O2—Si2	−51.9 (3)
Si11—Si1—O1—Al1	−145.50 (15)	K1—Al1—O2—Si2	−109.9 (2)
Si12—Si1—O1—Al1	97.37 (17)	Si31—Si3—O3—Al1	−62.1 (3)
K1—Si1—O1—Al1	160.1 (2)	Si32—Si3—O3—Al1	179.4 (2)
Si13—Si1—O1—K1	177.54 (4)	Si33—Si3—O3—Al1	54.6 (3)
Si11—Si1—O1—K1	54.39 (7)	O2—Al1—O3—Si3	−151.8 (3)
Si12—Si1—O1—K1	−62.73 (6)	O1—Al1—O3—Si3	73.8 (3)
O3—Al1—O1—Si1	98.69 (18)	Cl1—Al1—O3—Si3	−34.7 (3)
O2—Al1—O1—Si1	−35.5 (2)	K1—Al1—O3—Si3	22.7 (3)
Cl1—Al1—O1—Si1	−150.32 (16)	C111—Si11—C112—K1	−114.26 (11)
K1—Al1—O1—Si1	−159.9 (2)	C113—Si11—C112—K1	130.39 (12)
O3—Al1—O1—K1	−101.38 (8)	Si1—Si11—C112—K1	14.58 (11)
O2—Al1—O1—K1	124.41 (7)	C232—Si23—C231—K1^i^	115.2 (2)
Cl1—Al1—O1—K1	9.61 (6)	C233—Si23—C231—K1^i^	−129.0 (2)
Si23—Si2—O2—Al1	118.7 (2)	Si2—Si23—C231—K1^i^	−3.3 (2)
Si21—Si2—O2—Al1	−2.4 (3)	C312—Si31—C313—K1	110.5 (2)
Si22—Si2—O2—Al1	−119.5 (2)	C311—Si31—C313—K1	−134.0 (2)
O3—Al1—O2—Si2	63.4 (3)	Si3—Si31—C313—K1	−11.2 (3)
O1—Al1—O2—Si2	−162.4 (2)		

Symmetry code: (i) −*x*+1, −*y*+1, −*z*+1.
